# Dreaming during gastrointestinal endoscopy under propofol, ciprofol, or remimazolam anesthesia: study protocol for a parallel-design double-blind, single-center trial

**DOI:** 10.1186/s13063-023-07873-1

**Published:** 2024-01-02

**Authors:** Le-Qiang Xia, Rui Zhou, Rui Deng, Dan Zhou, Jia Han, Zhi-Fu Zhao, San-Jie Gao, Xian-Jie Zhang, Yu-Kai Zhou, Li-Ze Xiong

**Affiliations:** 1 Shanghai Key Laboratory of Anesthesiology and Brain Functional Modulation, Clinical Research Center for Anesthesiology and Perioperative Medicine, Department of Anesthesiology and Perioperative Medicine, Translational Research Institute of Brain and Brain-Like Intelligence, Shanghai Fourth People’s Hospital, School of Medicine, Tongji University, NO. 1279, Sanmen Road, Hongkou District, Shanghai, 200434 China; 2 Department of Anesthesiology, Deyang People’s Hospital, NO. 173, Section 1 of Taishan North Road, Deyang City, 618000 China

**Keywords:** Dreaming, Endoscopic sedation, Propofol, Ciprofol, Remimazolam

## Abstract

**Background:**

Dreaming sometimes occurs during sedation. It has been reported that factors such as different anesthetics, depth of anesthesia, age, sex, and preoperative psychological state may affect dreams. Ciprofol and remimazolam are novel choices for painless endoscopy. Herein, we aimed to investigate dreaming during gastrointestinal endoscopy under propofol, ciprofol, and remimazolam anesthesia respectively.

**Methods:**

This is a prospective, parallel-design double-blind, single-center clinical trial. Three hundred and sixty subjects undergoing elective painless gastroscopy, colonoscopy, or gastroenteroscopy will be enrolled. Eligible subjects will undergo propofol-, ciprofol-, or remimazolam-induced anesthesia to finish the examination. Interviews about the modified Brice questionnaire will be conducted in the recovery room. Incidence of dreaming is set as the primary outcome. Secondary outcomes include type of dreams, improvement of sleep quality, evaluation of patients, incidence of insufficient anesthesia, and intraoperative awareness. Safety outcomes are the incidences of hypotension and hypoxia during examination and adverse events during recovery.

**Discussion:**

This study may observe different incidences of dreaming and diverse types of dreams, which might lead to different evaluations to the anesthesia procedure. Based on the coming results, anesthesiologists can make a better medication plan for patients who are going to undergo painless diagnosis and treatment.

**Trial registration:**

This trial was registered at the Chinese Clinical Trial Registry on May 18, 2023 (registration number ChiCTR2300071565).

**Supplementary Information:**

The online version contains supplementary material available at 10.1186/s13063-023-07873-1.

## Introduction

Dreaming usually refers to the visual activity caused by various stimuli inside and outside the body or external stimuli remaining in the brain during sleep. As a part of consciousness, dream is an important neuroscientific issue. However, nightmares and undesirable dreams may induce by unpleasant experience and have an adverse impact on sleep quality and psychopathological wellbeing [1, 2]. Besides, the oddness may partly make dream an attraction to both scientists and everymen. Dreaming occurs in both natural and sedation-induced sleep. Are there any connections between anesthetics and dreams? It was reported that anesthetics could interfere with dreaming. Chen et al. [3] observed that dexmedetomidine preconditioning reduced the incidence of dreaming during general anesthesia. However, propofol was considered to contribute to the formation of dreams [4]. Previous studies indicated that approximately 20% to 40% of patients had a dream under propofol anesthesia [4, 5]. Moreover, dreamers induced by propofol were more satisfactory than non-dreamers, but this effect was not observed in midazolam-induced sedation [6].

Ciprofol is a new intravenous anesthetic that has a similar chemical structure to propofol. Ciprofol has some advantages over propofol. First, the potency of ciprofol is higher than that of propofol. Teng et al. [7] and Liu et al. [8] indicated that there were no significant differences in sedation or anesthesia induced by 0.4–0.5 mg/kg ciprofol and 2.0 mg/kg propofol. In other words, ciprofol is 4 to 5 times the potency of propofol. Second, the incidence of injection pain is much lower when compared with propofol [9, 10].

Remimazolam, a novel benzodiazepine sedative, can successfully induce general anesthesia [11]. The advantages of remimazolam are mainly reflected by its lighter suppression of the circulatory and respiratory systems [11–14]. Injection pain caused by remimazolam is also rare [12, 15]. Recently, remimazolam has been widely used for sedation and general anesthesia.

However, few studies have focused on the modulation of dreaming by ciprofol and remimazolam. We hypothesize that the three anesthetics have different influences on dreaming. Herein, we designed this clinical trial to research the influences of propofol, ciprofol, and remimazolam on dreaming.

## Methods and design

### Aim of this study

Anesthetics can affect the incidence and content of a dream. The aim of this study is to investigate dreaming during gastrointestinal endoscopy under propofol, ciprofol, and remimazolam anesthesia respectively. We hope to improve the experience of painless examinations by optimizing anesthesia medication.

### Study design and settings

**Fig. 1:**
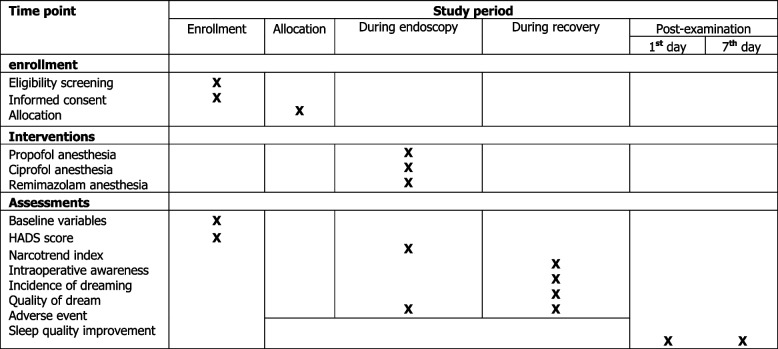
Schedule of this study. HADS, the Hospital Anxiety and Depression Scale

**Fig. 2:**
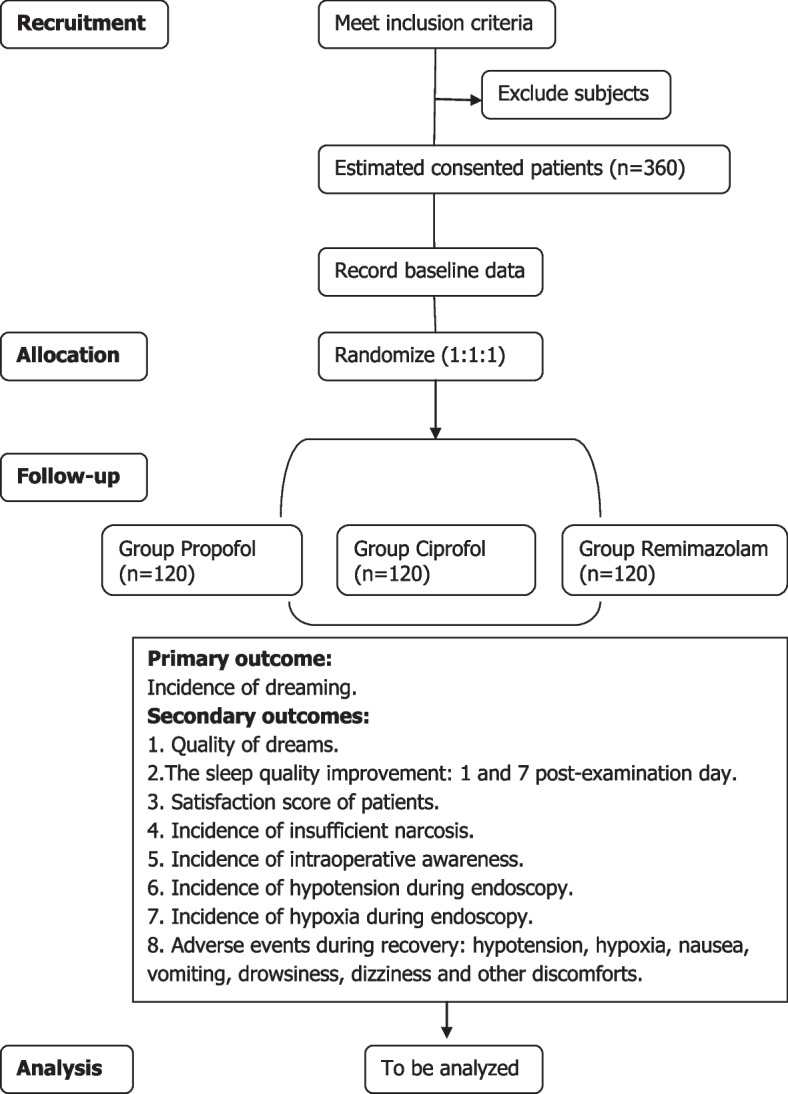
Study flowchart

This study is designed as a prospective, exploratory, parallel-design, double-blind, single-center clinical trial. The allocation ratio is (1:1:1). This trial was registered at the Chinese Clinical Trial Registry on May 18, 2023, and given the registration number ChiCTR2300071565. The protocol of this trial (version 2.1) is designed in line with the SPIRIT statement (Fig. 1) [16]. Figure 2 shows the study flowchart. The study is planned to be conducted in Deyang People’s Hospital, a comprehensive tertiary A hospital in China.

### Inclusion and exclusion criteria

Patients who meet the following criteria will be included: (1) Patients with an age of 18 to 65 years old, with American Society of Anesthesiologists (ASA) status I to II; (2) Patients with respiration rate from 12 to 18 cycles/min and SpO_2_ is more than 95% when inhaling air; (3) Patients whose heart rate is from 50 to 100 beats/min, systolic blood pressure is no less than 90 mmHg, and diastolic blood pressure no less than 60 mmHg; (4) Patients who plan to undergo elective painless gastroscopy, colonoscopy, or gastroenteroscopy; (5) Patients who are able to understand research process, communicate effectively with researchers, and sign informed contents.

The exclusion criteria are as follows: (1) history of allergy to the drugs or drug adjuvants, (2) contraindications to deep sedation or anesthesia, (3) opioid analgesic consumption within 1 month; (4) intake of more than 14 units of alcohol per week (1 unit is equal to 360 mL beer, 45 mL spirits with 40% alcohol, or 150 mL wine), (5) potential difficult airway, (6) pregnancy or lactation, and (7) serious psychological disorders.

When there are severe allergic reactions, adverse events, anesthesia accidents, or withdrawal of the subjects, the case will not be analyzed. Attending anesthesiologists will take charge of treating the patients who are with the abovementioned emergency events. These cases will firstly be reported to the head of department and then to the institution online.

### Ethical issues

The study was approved by the Ethics Committee of Deyang People’s Hospital on April 20, 2023, and the approval number is 2023-04-031-K01. Our investigators will introduce the study to eligible subjects and obtain the written informed consent. The subjects are allowed to withdraw from the study whenever they want. We declare that the study procedure will comply with the Declaration of Helsinki. The private information such as names will only be accessible to the investigators during the data collection period and be canceled when logging data to the database.

### Randomization, grouping, and blind method

A randomized sequence is obtained from SPSS 23.0 (IBM SPSS Statistics, USA) by an independent investigator. The numbers are stored in sealed opaque envelopes consequently. Once the first subject is enrolled, the anesthesiologist in the examination room will open the first envelope to read the grouping information. As the subjects are in the lateral position and vein access is sheltered, the subjects are blinded to the medication. The nurse anesthetist responsible for interviews and follow-ups will not participate in anesthesia management. Therefore, this is a subject- and observer-blinded study.

### Intervention

Enrolled patients will firstly complete the Hospital Anxiety and Depression Scale (HADS) survey [17]. Then, the nurse anesthetist will open vein access and guide patients to position themselves in a standard posture for gastrointestinal endoscopy. After getting into the examination room, noninvasive blood pressure, SPO_2_, electrocardiogram, and Narcotrend index will be continuously monitored. Oxygen with a flow of 8 L/min will be provided continuously by a ventilation device. Then, the subjects will be randomly allocated into the propofol group (group propofol), ciprofol group (group ciprofol), or remimazolam group (group remimazolam) according to the information in the envelopes. Anesthesia induction will be initiated by sufentanil (0.06–0.08 μg/kg, Yichang Humanwell Pharmaceutical Co., Ltd., China), followed by propofol (1.5–2 mg/kg, Yangtze River Pharmaceutical Group, China), ciprofol (0.3–0.5 mg/kg, Haisco Pharmaceutical Group, China) or remimazolam (0.2–0.3 mg/kg, Yichang Humanwell Pharmaceutical Co., Ltd., China). When the Narcotrend index turns to C, endoscopy is started by a skilled gastroenterologist. Before the Narcotrend index changes to B, top-ups will be administered. One bolus of top-ups is one-third to one-fifth of the loading dose.

If there is a hypotension, which is defined as systolic or diastolic pressure reduced by more than 20% of baseline, 2 mg dopamine (Wuhan Jiuan Pharmaceutical Co., Ltd., China) boluses will be used. Jaw thrust and maneuver ventilation will be performed to improve oxygenation when SPO_2_ is less than 90%. Heart rate between 45 and 50 beats per minute is unnecessary to treat as if the blood pressure is in the safety range, or 0.3 mg atropine (Anhui Changjiang Pharmaceutical Co., Ltd., China) blouses will be administered.

Patients will be transferred to the post-anesthesia care unit (PACU) for recovery. A nurse anesthetist who has been well trained and is blinded to the allocation will interview the patients. A modified Brice questionnaire will help to determine the incidence of dreaming and intraoperative awareness, as well as the quality of dream. Intraoperative awareness is recognized if the patient can recall the things that happened during the operation and can tell whether there is pain or not. Evaluation of anesthesia is represented by numbers from zero to ten. Zero is for extremely unsatisfied, while ten for extremely satisfied. Improvement of sleep quality at 1 and 7 post-examination days will be collected by telephone call of the nurse anesthetist. Patients are required to evaluate the improvement of sleep quality by yes or no according to their own feelings.

The modified Brice questionnaire consists of the following five questions [18]: (1) What is the last thing you can remember before falling asleep? (2) When you just wake up, what do you first remember? (3) What do you remember between them? (4) Do you dream during sleep? (5) What do you think about your dreams: pleasant, unpleasant, or neutral?

Adverse events, such as hypotension, hypoxia, nausea, vomiting, dizziness, drowsiness, and other discomforts, will be documented by the nurse anesthetist. Attending anesthesiologists will participate in the treatment of these side effects. Patients are allowed to go home with a companion if they are fully recovered from unawareness and unorientation, stable in hemodynamic and respiratory parameters, and free of nausea and vomiting.

### Outcomes

The primary outcome is the incidence of dreaming. Secondary outcomes are as follows:

1. Quality of dreams (during recovery): pleasant, unpleasant or neutral based on their own feelings;
2. Sleep quality improvement (1 and 7 post-examination days): “yes” or “no” according to their specific conditions;
3. Patient satisfaction score to anesthesia (at the end of recovery): zero to ten for extremely unsatisfied to extremely satisfied;
4. Incidence of insufficient anesthesia (during anesthesia): defined as Narcotrend index changes to A or B before finishing the endoscopy;
5. Incidence of intraoperative awareness (during recovery);
6. Safety evaluation (during anesthesia and recovery): incidence of hypotension and hypoxia during examination and adverse events during recovery. The classifications of adverse events during recovery include hypotension, hypoxia, nausea, vomiting, drowsiness, dizziness, and other discomforts.

### Data collection and management

In addition to the mentioned data, baseline data, including demographic data, HADS score, kind of endoscopy, time to adequate anesthesia, duration of examination, and consumption of anesthetics will also be collected. Preoperative, intraoperative, and postoperative data will be documented on three pieces of paper by different investigators. Namely, the nurse anesthetists are responsible for recording the preoperative and postoperative data, while anesthesiologists in the examination room for the intraoperative ones. All data will be input and stored in the SPSS software by an independent researcher. Professor Li-Ze Xiong, Xian-Jie Zhang, and Yu-Kai will take charge of managing the data. To improve data quality, all the researchers will be trained, such as the eligibility evaluation, randomization and blind protection, and interview skills.

### Sample size determination

Since there were few studies that reported the incidence of dreaming induced by ciprofol or remimazolam, we performed a preliminary study in which 20 cases were included in each group. The results showed that the incidence of dreaming was 25%, 45%, and 35% in group propofol, group ciprofol, and group remimazolam, respectively. Sample size was calculated by the Compare K Proportions model on *http://powerandsamplesize.com/*. Given a 0.05 type I error (*α*) and 0.8 power (1−*β*), 114 subjects were sufficient for each group to detect differences in dream incidence. Considering 5% dropout, we intended to recruit 360 subjects.

### Recruitment plan

Patients who plan to undergo a painless gastrointestinal endoscopy are required to have a preoperative evaluation of anesthesia. After a thorough evaluation, eligible patients will be asked if they would like to participate in this trial. The study protocol, risks, and benefits will be introduced to the patients. The related documents were reviewed and approved by the Ethics Committee of Deyang People’s Hospital. No persuasive advertising will be used to help with recruitment. To promote participant retention and completion of follow up, we will inform them that the time points of phone calls during recruitment period and reserve at least 2 records of contact number. Recruitment were planned to begin on May 21, 2023, and end on October 10, 2023.

### Statistical analysis

SPSS 23.0 software will be applied for statistical analysis. Count data will be presented as case number or percentage. The chi-square test will be used to analyze the differences among or between the groups. For measurement data, the Kolmogorov–Smirnov test will be applied to identify the distribution of data. Data obeyed normal distribution are depicted as the mean ± standard deviation (SD). One-way analysis of variance (ANOVA) will be used to test the differences among the groups. LSD-*t* test will be responsible for pairwise analysis. Abnormally distributed data will be shown as the median (inter-quartile range) and analyzed by the Kruskal–Wallis test, while the Wilcoxon test is for pairwise comparisons if necessary. A *P* value less than 0.05 with two tails is regarded as statistically significant. For pairwise comparison with chi-square test, the significant level of this study is adjusted to be 0.017. Additional analyses such as subgroup and adjusted analyses will not be conducted. Considering primary data are obtained in hospital and the dropout rate is estimated to be low, the missing data of sleep quality will be not complemented. Interim analyses will not be applied, because the three anesthetics have passed the safety examinations before introducing to hospitals.

## Discussion

In recent years, the amount of painless gastrointestinal endoscopy has been increasing. It was reported that approximately one-fifth to one-quarter of them experienced dreams during painless gastrointestinal endoscopy [5, 19, 20]. Xu et al. [20] observed that among the dreamers, approximately one-third reported pleasant dreams. Studies have demonstrated that the main factors influencing dreams during anesthesia include the kind and dosage of anesthetics, depth of anesthesia, sex, and preoperative psychological pretreatment [4, 5, 20–23]. Furthermore, Yoshida et al. [24] found that a less than 11 depression score of the HADS was highly correlated with positive dreams.

In the present study, we intended to investigate dreaming during painless gastrointestinal endoscopy under propofol, ciprofol, and remimazolam anesthesia respectively. The primary aim of this study is to analyze the incidence of dreaming in the three groups. Since the duration of examination and recovery is short, we did not choose complicated scales. Instead, we selected the modified Brice questionnaire which has been widely applied by researchers to determine whether there is a dream or intraoperative awareness [3, 25, 26]. For those who have a dream, patients simply need to tell pleasant, unpleasant, or indifferent to evaluate the quality of the dreams.

To minimize the mentioned confounding factors, we plan to take some measures. First, we will test the HADS for the patients. In this way, some patients with severe undiagnosed anxiety and depression can be excluded. Second, the Narcotrend index will be monitored for its good consistency between sedation depth and propofol or benzodiazepines [27, 28]. Considering that the stimulus intensity of gastrointestinal endoscopy is relatively mild, sufficient anesthesia is defined as grade C of the Narcotrend index. This is similar to a previous study in which no intraoperative awareness was observed even though quite a few Narcotrand values were above 70 [29].

There are some limitations in the study. On the one hand, we do not prescribe a limit to the category of endoscopy. Gastroenteroscopy is more likely to take more time and drugs than gastroscopy. However, these two factors are not the outcome parameters. On the other hand, we do not administer the anesthetics in a continuous way, which may cause fluctuations in sedation. Since it is difficult for us to predict the duration of endoscopy, continuous administration may lead to explosive suppression of the brain. In addition, this is a single-center trial, and multicenter studies are still needed.

## Conclusion

In conclusion, this is a clinical trial aimed at investigating the influences of anesthetics on dreaming during painless gastrointestinal endoscopy. The results of this study may provide a better choice of medication for anesthesiologists and further improve the experience of patients undergoing painless endoscopy.

### Trial status

The first subject was recruited on May 21, 2023. The last one was enrolled on October 10, 2023. The latest protocol (version 2.1, September 12, 2023) in which a clerical error was revised was approved on September 26, 2023. Trial registration revision was finished on October 8, 2023. Considering revision of the protocol will not impact subject enrollment and analyses, previously recruited patients will not be excluded.

### Supplementary Information

**Additional file 1.**

## Data Availability

There are no specific data in this paper. Results of this trial can be obtained from Li-ze Xiong (mzkxlz@126.com, lizexiong@tongji.edu.cn) for reasonable requests.

## Abbreviations

ASA: American Society of Anesthesiologists
SpO_2_: Pulse oxygen saturation
HADS: Hospital Anxiety and Depression Scale
PACU: Post-anesthesia care unit
